# A new look at old compounds

**DOI:** 10.18632/aging.100317

**Published:** 2011-04-20

**Authors:** Silvestre Alavez, Gordon J. Lithgow

**Affiliations:** Buck Institute for Research on Aging, 8001 Redwood Blvd, Novato, CA 94945, USA

Aging is the single most important risk factor in human disease in developed countries but when it comes to research on prevention or cures, aging is seldom taken into account. Nevertheless if aging is a significant contributor to age-related conditions, we would hope that an understanding of aging mechanisms could prompt the design of rational therapies. Moreover, if aging causes multiple diseases then it is reasonable to think that pharmacological agents that slow aging could be also effective in preventing or slowing a wide spectrum of diseases.

Considerable progress has been made in understanding the genetic influences on lifespan in simple metazoans. In particular, the longevity of the nematode *Caenorhabditis elegans* (*C. elegans*) is influenced by hundreds of genes and metabolic processes including an insulin signaling-like (ISL) pathway that regulates the FOXO-like transcription factor DAF-16 [1(]. The ISL influences many processes including development, fertility and stress resistance. Its influence on the ability to withstand stress presents a connection to disease that could be exploited pharmacologically. In concert with the stress response transcription factor, Heat Shock Factor 1 (HSF-1), DAF-16 regulates the formation of toxic protein aggregation, reduces the sensitivity to stress and increases lifespan in *C. elegans* [2, 3]. This points to a critical role for protein aggregation in aging.

Protein aggregation is a hallmark of aging and several age-related pathologies, collectively known as conformational diseases (CD) [4]. This similarity strongly suggests a crosstalk between aging and disease. Although it is not clear how protein aggregation occurs, dramatic alterations in the balance of protein synthesis, protein folding and protein degradation (together representing “protein homeostasis”) are likely to play important roles in this process. As a consequence, modified proteins tend to accumulate into soluble oligomers and insoluble aggregates that may actively influence cell function. Neurodegenerative diseases are arguably the best studied CD and the aberrant aggregation of several insoluble molecules like α-synuclein (Parkinson's), β-amyloid (Alzheimer's) and huntingtin (Huntington's) has long been associated with the development of these pathologies [5]. However, other non-neurological, systemic diseases like type II diabetes and several myopathies, are also conformational diseases.

The general picture that that has emerged is that conformationally-altered proteins escape the surveillance of repair and degradation systems, form aggregates, and this process contributes to aging; aging could be therefore a manifestation of a loss in protein homeostasis. This then prompts the question: to what extent could chemical modulation of protein aggregation alter the rate of aging? Furthermore, would such an intervention influence disease pathology?

In a recent publication, we addressed this issue by identifying small molecules able to slow protein aggregation in the *C. elegans* model. We were then able to directly assess the degree to which protein aggregation influences normal aging rates [6]. We undertook a focused screen of chemical compounds that exhibit protein aggregate-binding properties, such as Thioflavin T (ThT) and curcumin, widely used to stain these aggregates in post-mortem tissues of neurological disease affected individuals. We found that exposing wildtype worms to 50 μM ThT throughout the adult life leads to an increase in median and maximal lifespan and reduced age-specific mortality at all ages. The compound also slowed age-related decline in spontaneous movement throughout adulthood which generally indicates improved health. However, at higher concentrations (500 μM), ThT was clearly toxic. Whether this is the result of “off target” side effects or something specific to protein aggregation is not clear. Interestingly, we found that several compounds structurally related to ThT are also able to increase lifespan suggesting that general structural features of ThT are important for lifespan extension in C. elegans. This result is of particular interest because it implies the possibility that other ThT structurally-related compounds with different bioavailability and physical chemistry properties could be used with similar success to increase lifespan or ameliorate conformational diseases.

Several models of protein aggregation of the type observed in neurodegenerative diseases have been developed in C. elegans. Elegant work from the laboratories of Richard Morimoto [7] and Christopher Link [8], among others, has provided the worm community with robust models of protein aggregation that we exploited. We tested whether ThT and other compounds could also prevent protein aggregation-associated phenotypes. Indeed, they did. We then used another wonderful worm tool, RNA interference, to ask whether compound action required endogenous worm factors, particulary components of the protein homeostasis network. In short, these experiments told us that that ThT requires an intact protein homeostasis network to produce beneficial effects on these strains. In addition, we found that the ThT effect on lifespan depends of two transcription factors that have long been associated with the control of the stress resistance and longevity, Heat Shock Factor 1 (HSF-1) [3] and SKN-1 [9].

A large body of literature demonstrates that ThT binds to amyloid-like aggregating protein [10]. This suggested to us that a molecular interaction of the compound with misfolded proteins could be occurring in vivo. Although we were unable to clearly demonstrate this interaction in worms engineered and induced to express amyloid for 24 h we were able to visualize ThT in cells containing large aggregates. We believe that ThT could exert its influence on protein homeostasis initially by binding to aberrant forms of protein but may also be interacting with protein homeostasis regulatory targets or transiently binding to the amyloid itself at early times after amyloid induction.

These results indicate that the protein homeostatic network can be manipulated by using small molecules to suppress age-related disease pathologies and highlights some important concepts for a novel approach to the discovery of new drugs with potential to modulate aging and improve the conditions of conformational diseases.

We hypothesize that compounds that enable an animal to maintain protein homeostasis during aging could be active against multiple states of diverse conformational diseases. These compounds can be identified by using *C. elegans* models of neurodegenerative disease and aging. Perhaps prevention and cures of age-related diseases will emerge from chemical screens that target aging.
